# ACID: a free tool for drug repurposing using consensus inverse docking strategy

**DOI:** 10.1186/s13321-019-0394-z

**Published:** 2019-11-27

**Authors:** Fan Wang, Feng-Xu Wu, Cheng-Zhang Li, Chen-Yang Jia, Sun-Wen Su, Ge-Fei Hao, Guang-Fu Yang

**Affiliations:** 1 Key Laboratory of Pesticide & Chemical Biology, Ministry of Education, College of Chemistry, Central China Normal University, Wuhan, 430079 People’s Republic of China; 2 International Joint Research Center for Intelligent Biosensor Technology and Health, Central China Normal University, Wuhan, 430079 China; 3 State Key Laboratory Breeding Base of Green Pesticide and Agricultural Bioengineering, Key Laboratory of Green Pesticide and Agricultural Bioengineering, Ministry of Education, Research and Development Center for Fine Chemicals, Guizhou University, Guiyang, 550025 People’s Republic of China; 4 Collaborative Innovation Center of Chemical Science and Engineering, Tianjing, 300072 People’s Republic of China

**Keywords:** ACID, Web server, Drug repurposing, Consensus inverse docking

## Abstract

Drug repurposing offers a promising alternative to dramatically shorten the process of traditional de novo development of a drug. These efforts leverage the fact that a single molecule can act on multiple targets and could be beneficial to indications where the additional targets are relevant. Hence, extensive research efforts have been directed toward developing drug based computational approaches. However, many drug based approaches are known to incur low successful rates, due to incomplete modeling of drug-target interactions. There are also many technical limitations to transform theoretical computational models into practical use. Drug based approaches may, thus, still face challenges for drug repurposing task. Upon this challenge, we developed a consensus inverse docking (CID) workflow, which has a ~ 10% enhancement in success rate compared with current best method. Besides, an easily accessible web server named auto in silico consensus inverse docking (ACID) was designed based on this workflow (http://chemyang.ccnu.edu.cn/ccb/server/ACID).

## Introduction

In recent years, the productivity challenge facing the pharmaceutical industry has become particularly difficult to overcome [1]. By many estimates, the number of new molecular entity approved to market per billion US dollars spent on (research and development) R&D has halved roughly every one decade, falling around 80‑fold in inflation-adjusted terms [2]. To increase drug-discovery productivity, more and more attention has been paid to exploring the relationship between drug and disease, which can advance our knowledge of molecular mechanism of disease indication and lead to new strategies to treat productivity challenge [3, 4]. Nevertheless, traditional strategies which typically oriented on a search for a novel therapeutic compound combined with discovery of a new therapeutic target are time consuming, expensive and risky because of the necessity for multiple experimental and clinical validation [5].

Drug repurposing/repositioning/rescue, the application of an existing drug to a new disease indication, is a promising approach to address the ‘productivity gap’, especially the demand of rapid clinical impact at a lower cost by the ‘starting-from-scratch’ drug development [6]. Compared with brand new drug discovery for a given disease indication, this method has several advantages. First, due to the existing drug has already been proved to be sufficiently safe in humans, the safety risk of clinical failure is much lower at least from a safety point of view. Second, due to the safety assessment and most of formulation task have already been completed, the development cycle should be largely reduced. Third, the investment is always less [7]. These advantages have made the development of repurposed drugs into a task of low risk investments with faster and higher returns. Hence, Drug repurposing is drowning widespread attention from the pharmaceutical industry, government agencies and academic institutes, such as ‘Discovering New Therapeutic Uses for Existing Molecules Plan’ by NIH (USA). However, drug repurposing is vastly more complicated than typically imagined and to date there has not been a systematic approach to identify repurposing opportunities.

In order to reduce the number of “wet” experiments and thereby reduce cost, extensive research efforts have been directed toward developing computational (virtual or in silico) approaches, which have been proved extremely valuable in identifying potential opportunities in these fields. Of the several techniques for generating computational repositioning hypotheses, inverse/reverse docking, involved docking an existing drug in the potential binding cavities of a set of clinically relevant disease targets, is proving to be a powerful tool for drug repositioning [8, 9]. Inverse docking is ‘one ligand-many targets’ scenario, representing a structure-based computational strategy. Different with the conventional drug virtual screening, inverse virtual screening was performed for a small-molecule against a large collection of binding-sites of clinically relevant macromolecular targets. The top-ranking targets based on the binding complementarity (shape and electrostatics) with the drug are likely to result in potential drug repositioning. Hence, efficient tools were developed for inverse docking, for example, INVDOCK [10], TarFisDock [11], PDTD [12], and idTarget [13]. Moreover, successful drug repurposing examples along with these tools are steadily grows, such as sildenafil and thalidomide [14]. Since the basic philosophy behind reverse docking is the same with docking and the critical parameters of the docking programs were always optimized based on some of the specific ligand and target systems, the performance in docking pose search itself and scoring of the docked poses may, thus, still face challenges for reverse docking methods. Up to date, many studies have proved that the consensus strategy that combining several types of docking algorithm can achieve higher success rates in pose prediction than single docking algorithm [15]. Hence, development of consensus inverse docking algorithms to address the inherent difficulties involved in the molecular docking, is extremely valuable in identifying potential opportunities of drug repurposing [9]. In addition, due to that almost all current docking tools are designed for ‘one ligand-many targets’ scenario, the usability of tools for inverse virtual screening task is occasionally restricted by code-writing dependencies and tedious operation steps, which bring challenges for non-expert users. Therefore, there is still a strong demand for a new free server of inverse docking.

Hence, we developed a computational protocol by combining the results of several dissimilar types of free docking method into a consensus inverse docking (CID) scheme. Here, we selected AutoDock Vina, LEDOCK, PLANTS, and PSOVina for binding pose search as they represent significantly different docking methodologies (i.e., different conformational search algorithm, different global and local optimizers, and different scoring functions) and have employed different collections of crystal complexes and binding data to calibrate their optimization algorithms. In addition, we used Molecular Mechanics/Poisson–Boltzmann Surface Area (MM/PBSA) and X-SCORE for final binding energy calculation as they are more rigorous than the intrinsic scoring function in principle. The intention was to investigate whether integration of these to develop a consensus strategy to the inverse docking problem would result in improvements in posing accuracy and prediction of binding modes. Besides, in order to significantly reduce user time for data gathering and multi-step analysis for drug repurposing task, an comprehensive web platform named Auto in silico consensus inverse docking (ACID) (http://chemyang.ccnu.edu.cn/ccb/server/ACID) with a user-friendly interface was also designed for an easy evaluation and application of this strategy, which consists of the following three tools: (i) an automated consensus inverse docking workflow program, (ii) a compound database containing 2086 approved drugs with original therapeutic information, (iii) a known target database containing 831 protein structures from PDB covering 30 therapeutic areas.

## Methods

### Selection of test set

The PDB-bind database is a large collection of protein−ligand crystal complexes with associated experimentally determined binding data [16]. A total of 16,151 protein–ligand entries are contained in the PDB-bind database. In all protein–ligand entries, there are 4463 entries with good quality structural and binding data. The experimental resolution of any chosen crystal structure should be lower than 3.0 Å, because adopting structures with poor resolution may generate false predicted conformations [17]. No NMR-solved structures were selected in our benchmark dataset. Any complexes with ligands containing off-standard atom types (like Si or Be) were rejected. Finally, we only chose complexes with a single drug like molecule bound in the active site. Hence, a subset contains 195 complex structures of these have been selected as the “core set”, aiming to provide high-quality complexes that represents a broad cross-section of the database. This is of appropriate size for evaluation studies of docking and scoring performance [18].

### Preparation of structures for inverse docking

For each complex, the original conformation of ligand was extracted from the PDB file; similarly, the 3D structure of corresponding protein was generated. As for the proteins, water molecules were removed and the program PDB2PQR 1.6 21 was used to assign position-optimized hydrogen atoms under the protonation state simulated to pH = 7.0. The AMBER ff14SB [19] force field were adopted for charge assignment. The MOL2 format representation of each receptor was prepared by SPORES tool in Plants software package. The Autodock Tools 1.5.4 utility prepare_receptor4.py was used to assign Gasteiger charges to atoms. The Autodock Tools utility prepare_ligand4.py was used to assign Gasteiger charges and rotatable bonds. The input grid files were prepared by dms and sphgen_cpp tools of DOCK software package.

### Selection of docking softwares

Seven docking softwares were carefully evaluated to build consensus strategy, including AUTODOCK [20], VINA [21], DOCK [22], PLANTS [23], PSOVINA [24], LEDOCK (http://www.lephar.com) and GOLD [25]. This selection covers a wide variety of conformation search algorithm and scoring function (Additional file 1: Table S1), thus representing an abundant source for optimizing the consensus protocol. The docking calculation was performed on the prepared dataset of 195 receptors and ligands by using these seven docking softwares based on default parameters. The box within the surrounding 12.5 Å of the bound ligand was defined as active site. 100 conformations for each ligand versus its corresponding active site were produced by each software. The one with highest score were selected as the final pose.

### Binding free energy calculation

Before binding free energy calculation, the Sander module in Amber16 [26] program was used to perform the three-step optimization of the ligand-receptor complex. Firstly, only waters, ions and hydrogens were allowed to move. Secondly, the backbone atoms of the protein were fixed while others were allowed to move. Thirdly, all the atoms of the system were free to move. In the three optimization process, 2000 steps steepest descent method followed by 2000 steps conjugated gradient method were used for each ligand-receptor binding system. Finally, the binding free enegy (Δ*G*_bind_) is calculated by using the MM/PBSA [27, 28] and X-score methods [29, 30].

As for the X-score method, it is assumed that the overall binding free energy in a protein-ligand binding process can be divided into several terms (shown in Eq. 1) [31]. Here, Δ*G*_vdw_ represents the van der Waals interaction between the receptor and the ligand; Δ*G*_H-bond_ represents the hydrogen bonding between the receptor and the ligand; Δ*G*_deformation_ represents the deformation effect; Δ*G*_hydrophobic_ represents the hydrophobic effect; Δ*G*_0_ represents a regression constant. Δ*G*_bind_ value between the receptor and ligand could be calculated simply by the X-score software package.

1 $$\Delta G_{\text{bind}} = \Delta G_{\text{vdw}} + \Delta G_{{{\text{H}} - {\text{bond}}}} + \Delta G_{\text{deformation}} + \Delta G_{\text{hydrophobic}} + \Delta G_{0}$$

In the MM/PBSA method [32], the free energy of the receptor/protein-inhibitor binding, ∆*G*_bind_, is obtained from the difference between the free energies of the receptor/protein-ligand complex (*G*_cpx_) and the unbound receptor/protein (*G*_rec_) and ligand (*G*_lig_). The binding free energy (Δ*G*_bind_) was evaluated as a sum of the changes in the binding energy (Δ*E*_bind_), solvation entropy (−*T*Δ*S*_sol_), and conformational entropy (−*T*Δ*S*_conf_) (shown in Eq. 2) [33]. Where ∆*E*_bind_ is interaction energies between a ligand and a protein, which were computed using the Sander modules of the Amber16 program. The entropy contribution to the binding free energy (−*T*∆*S*) was obtained by using a local program developed in our own laboratory [33].

2 $$\Delta G_{\text{bind}} = \, \Delta E_{\text{bind}} {-}T\Delta S_{\text{sol}} {-}T\Delta S_{\text{conf}}$$

### Server implementation

As shown in Additional file 1: Figure S1, ACID web server mainly consists of three parts: Model (M), View (V) and Controller(C). Model is an object, which can provide a series of convenient APIs to access database. View represents the web service available for users. Controller is a Perl script program to control the whole consensus inverse docking protocol. A dedicated Linux machine in the high-performance computer cluster is used to run ACID web server. PHP (version 5.6), Apache (version 2.0.51), HTML5 and Javascript are used in the web application to provide online web service. The web server was established in the ‘Linux + Apache + MySQL + PHP + Javascript’ framework. The web interface is written in JavaScript using the React.js (input page, output page, and dataset visualization) and Ember.js (results and analysis pages) frameworks. The server side is written as a Python Cornice Web Framework with a GO component for rapid searching. A Torque management system is used to queue submitted jobs. The database which stores corresponding messages and results of each task is implemented by using MySQL (version 5.1.73). Results are stored for one month before deletion. The JSmol interactive molecular viewer plugin (http://www.jmol.org/) is applied in structure visualization. Firefox or Chrome explorer is recommended for browsing the server. Computer Screen with resolution higher than 1440 × 900 is recommended for displaying the web pages.

## Results and discussion

### Consensus inverse docking strategy

Consensus strategy may have relatively higher pose prediction performance than single docking software [34]. Hence, to select suitable docking methods to construct consensus inverse docking protocol, conformation prediction performance of these softwares was carefully evaluated. The final pose of each software was selected according to docking score. The RMSD value between each pose and its original conformation in complex crystal were calculated. If the RMSD < 2.0 Å, the corresponding pose prediction was success. The testing results are shown in Additional file 1: Figure S2. In our benchmark, GOLD software with GoldScore showed a slightly higher accuracy 66%. Taking the commercial copyright restriction into account, four academically free softwares, including VINA (63%), PLANTS (62%), PSOVINA (64%), and LEDOCK (64%) were selected to construct the consensus inverse docking method.

**Fig. 1:**
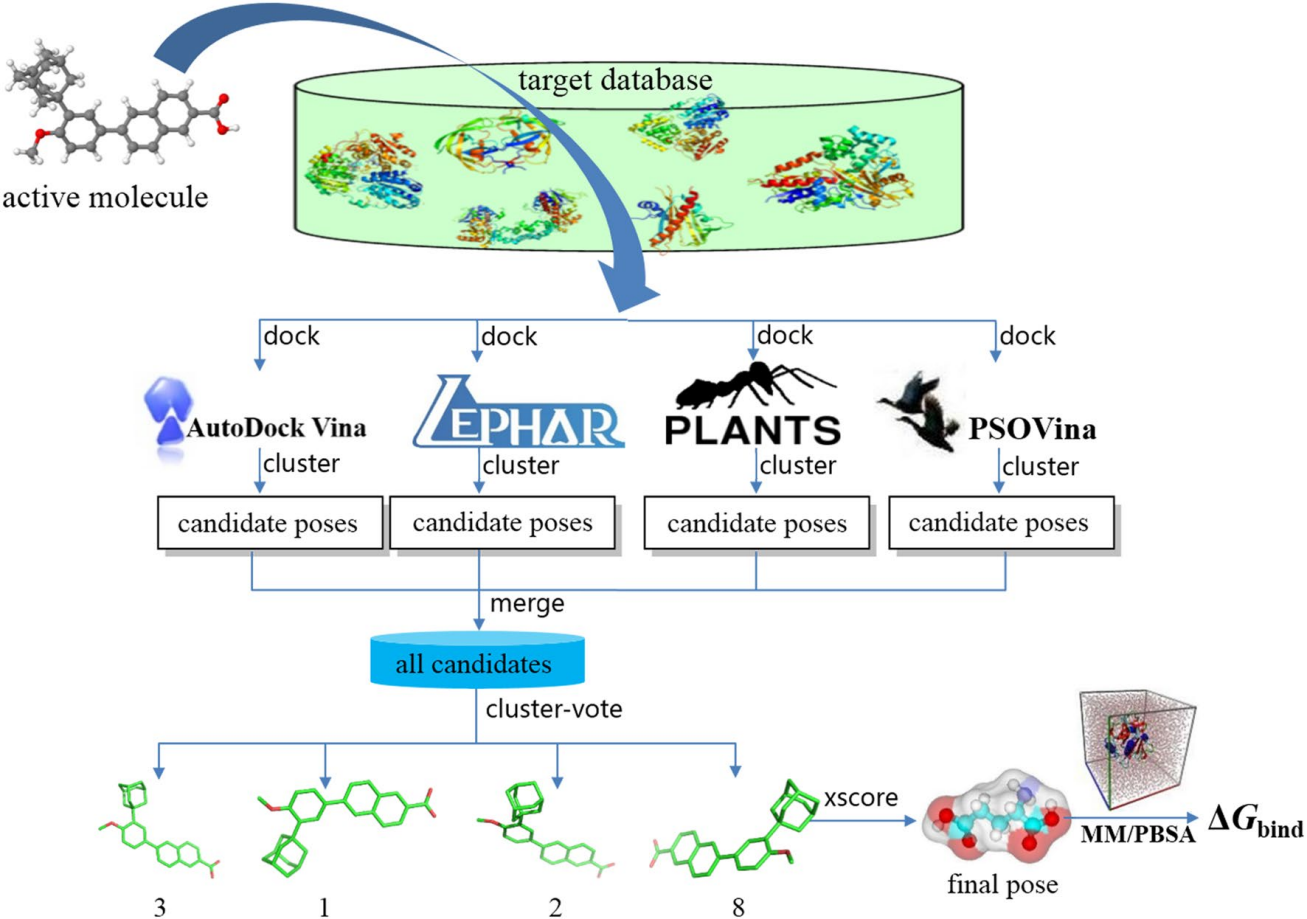
Workflow of consensus inverse docking protocol. The arrows denote the computational process

The consensus strategy can simulate a real-life voting process, because a wide range of voting processes can effectively avoid mistakes in decisions. Hence, a well-designed conformational *cluster*-*vote* strategy was optimized by using these four softwares (shown in Fig. 1). The detailed process of CID is like the following: First, initial 3D conformations of the given active molecule was generated and optimized by using MMFF94 force field [35]. Secondly, the optimized conformation was docked into the active site of each protein and four subsets of docking conformations were produced, which contain the poses independently predicted by a certain docking program. Third, the conformational clustering is performed in each conformational group. We calculate the RMSD value between each pose and create a similarity matrix in a conformational group. The conformations with RMSD value lower than 2.0 Å can be considered as the same conformational cluster. The *vote* value of each conformational cluster is equal to the pose number in this cluster. The conformation with best score can be used as a representative of this conformational cluster. Finally, the number of conformational cluster of each individual docking method could be obtained.

A set of representative conformations from each docking algorithm were selected to efficiently inspect different guided search algorithms for correct conformation of a protein–ligand complex. The representative conformation of each conformational cluster from the four docking methods was used to make up a new conformational ensemble, and then the same clustering method is performed to select the strongly binding conformations. The number of conformation is the number of votes. For example, if a conformation cluster from LEDOCK was also predicted by PSOVina, Vina, and PLANTS, that has the RMSD value lower than the threshold value of 2.0 Å, such a conformation cluster is qualified as 4 votes. The higher the vote number, the higher the support rate of the conformational cluster. In the case of vote dataset, the highest quality predicted conformation cluster has 4 votes. However, if there are two or more top clusters have the same votes, the pose number obtained by each docking method will be taken into account to judge. Finally, the highest vote conformational cluster was used to perform binding affinity calculation with X-score and MM/PBSA methods [29, 30].

### Performance and comparison with existing tools

A collection of target structures with the information of approved therapeutic drugs and potential ligand binding cavities is the prerequisite of drug repurposing. Since this database is used to search the probable binding proteins for existing drugs by using inverse docking, it only contains the proteins with 3D structures. The target proteins were selected from several online databases such as DrugBank [36], Uniprot [37], and PDB [38]. In order to integrate with consensus inverse docking protocol, drug target structure database should be constructed to store each protein in both PDB format and mol2 format with basic information, including docking parameters and active site information. Finally, we collected a database of experimentally confirmed 831 drug targets and 2086 drug compounds. To evaluate the performance of CID protocol, the screening was performed on the 831 experimentally confirmed drug targets and 51 out of the 2086 collected commercial drugs were selected to compose a test set according to the two criterias. (1) The cocrystal structures of the drug and its targets should be available. (2) The drugs in the test set should have a wide representation of the whole commercial drug dataset. The ligand flexibility, which can be assessed by the number of rotatable bond, can have a major impact on the performance of docking method. According to number of rotatable bonds, the distribution of whole commercial drug dataset and 51 sampled drugs were quite similar (shown in Additional file 1: Figure S3), which demonstrated that our testing samples have a wide representation.

**Fig. 2:**
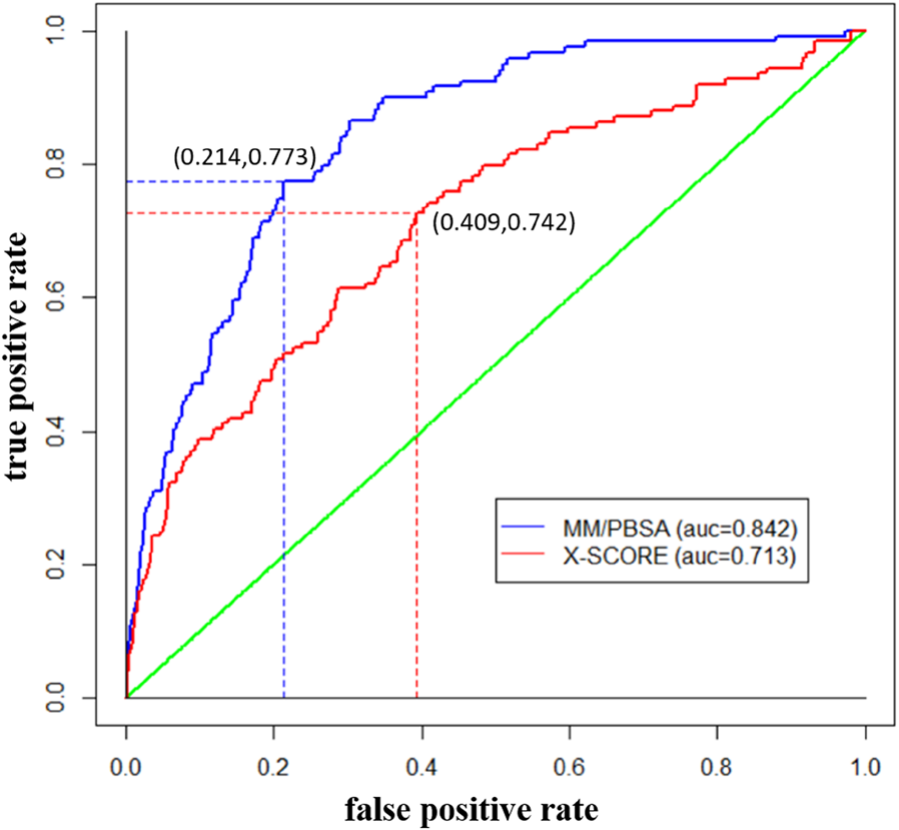
The specificity and sensitivity of ACID performance on distinguishing target and non-target of drugs based on MM/PBSA and X-Score

To analyze of the performance of the prediction, we performed the receiver operating curve (ROC) analysis [39], which is a graphical plot to illustrate the diagnostic ability of a binary classifier system. According to its discrimination threshold, the area under the ROC (AUC) values can be used to evaluate the ability to distinguish between target and non-target. The known targets of each drug were considered as positive samples, the other proteins in our screening dataset were considered as negative set. Although it does not eliminate the possibility that some of them may interact with these drugs, the number of this kinds of protein is less. In our performance test, ROC analysis was used to compare the performance of the prediction between MM/PBSA and Xscore. The AUC was computed based on the following: If the true targets are ranked in the top 10%, it was considered as true positive. If the true targets are ranked out side of the top 10%, it was considered as false negative. If the non-targets are ranked in the top 10%, it was considered as false positive. If the non-targets are ranked out side of the top 10%, it was considered as true negative. Figure 2 shows the ROC curves and the AUC, which is used to assess the discriminative performance of MM/PBSA (AUC = 0.842) and XScore (AUC = 0.713). It shows that MM/PBSA outperforms XScore in our performance test, which could be due to the nature of the general applicability and the universal physical scale of the energy calculation methods [40]. In addition, we also retain a higher false positive rate for MM/PBSA (21.4%) and Xscore (40.9%). It may probably due to that the number of negative sample is much larger, hence even a low error rate of negative samples may cause many false positive predictions.

The binding free energies calculated by MM/PBSA method were further analyzed to examine the binding of a variety of proteins to drug (listed in Additional file 1: Table S2). The “TRUE” value under “2%, 5%, and 10%” column means that the known targets of the testing drug are identified in the top 2%, 5%, and 10% of the corresponding results. While “FALSE” means the known targets are identified out of the top 10%. As shown in Additional file 1: Table S3, 35 assessed drugs showed significant enrichment of their known targets in the top 10%. In addition, 18 assessed drugs were identified in the top 2%. Therefore, the top 2%, top 5% and top 10% prediction success rate were 35.29%, 52.94% and 68.63% respectively. Taking the tricyclic antidepressants Amitriptyline as an example, 9 known targets out of 11 extracted from literatures and other databases was identified in top 10%. 5J03 appeared among top 11% ranked proteins. 4PMP is a false negative as it did not appear among the 100 best ranked structures.

**Fig. 3:**
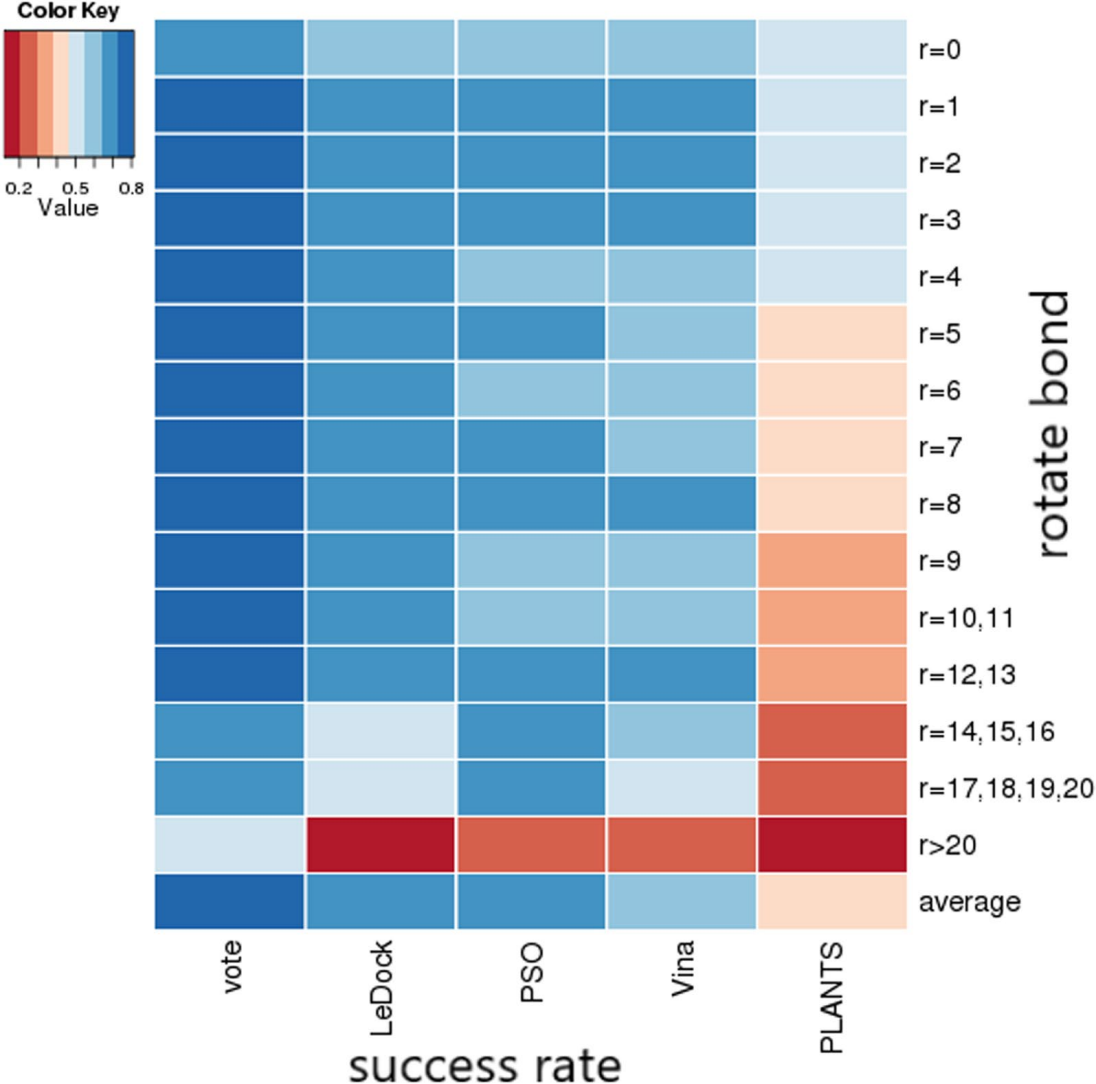
Pose prediction performance of consensus inverse docking and individual methods

In order to evaluate if there is an improvement, the conformation prediction performance of CID protocol was compared with individual docking method. According to the criterion of top 10%, CID protocol showed significant higher pose prediction performance (74.4%), which is around 10% improvement in comparison with the best result obtained from LEDOCK (64%). The prediction performance of other individual docking method is 64% (PSOVina), 63% (Vina), 42% (PLANTS), which are statistically analyzed according to the successfully predicted drugs of 30 in LEDOCK, 30 in PSOVina、29 in Vina, 20 in PLANTS. In addition, it is important to evaluate that if the docking accuracy can be improved on ligands with more rotatable bonds. The novel CID protocol showed a higher successful rate in a wider range of rotatable bonds compared with any individual docking method. The distribution of the docking results according to the number of rotatable bonds are shown in Fig. 3. Therefore, it was proved that CID protocol may have relatively higher prediction performance than these single docking methods.

Meanwhile, we also compared the prediction performance of ACID with other drug repurposing prediction tools. Due to the different requirement in drug repurposing study, there was not a uniform standard or a same dataset to evaluate drug repurposing prediction performance. Hence, we use several criterion like AUC and TOP as indicators to evaluate the predictive performance of these studies. Compared with other drug repurposing prediction tools, the AUC indicator of ACID is 0.84, which is a little lower than idTarget. But the sample set of ACID is 51 drugs and 91 known targets, which much larger than idTarget and TarFisDock. In addition, ACID can find 62 known targets in Top 2%, 76 known targets in Top 5%, and 91 known targets in Top 10%, which is better than TarFisDock. Compared with similarity comparison based approaches, the prediction accuracy of ACID are still dominant. However, due to the nature of structural comparison between small molecules, the similarity comparison based approaches may offer advantages such as faster computations and a larger tested sample number. While, docking based approaches includes three dimentional structures, structural optimization, conformational search, and binding energy calculation, which would obviously increase the computational cost. Thus the test sample set is much smaller than similarity approach. However, they can potentially identify novel targets for the drug which may be relevant for its mechanism of action or side effect profile. Based on the above analysis, we can infer that ACID keeps better or comparative predictive ability compared with similar tools. The detailed methods, sample sets, and accuracy of comparison were summarized in Table 1 [10, 11, 13, 41–44].

**Table 1:** Several drug repurposing tools compared with ACID

Name	Method	Sample set^a^	Prediction performance		Date of last update	Refs.
			AUC^b^	TOP(2%/5%/10%)^c^		
Similarity comparison based approaches						
ChemMapper	3D similarity approach	216/7069	0.7	–	Dec 2016	[25]
ChemProt 3.0	2D similarity approach	248/1700	0.827	–	Jan 2015	[26]
HitPick	1NN similarity search approach	3430/3116926	0.61^d^	–	May 2013	[27]
SwissTarget-prediction	Combination of 2D and 3D similarity approach	346/1730	0.87	–	Apr 2014	[28]
Docking algorithm based approaches						
idTarget	Divide and conquer based docking approach	1/3/1161, 1/4/1161	0.89, 0.91	–	Aug 2015	[13]
INVDOCK	Inverse docking approach	2/23/2700	–	50%^e^	May 2001	[10]
TarFisDock	Reverse docking approach	1/10/37, ··· 1/12/371	–	33%/33%/58%, 30%/20%/50%	Aug 2014	[11]
ACID	Consensus inverse docking approach	51/133/831	0.84	47%/57%/68%	Dec 2018	

^a^The sample set is number of positive/negative interactions for the similarity comparison based approaches, and is number of drugs/known targets/decoys for docking algorithm based approaches

^b^The AUC (Area Under Curve) is used to represent the prediction performance in the references cited, the closer the AUC value is to 1, the better the prediction performance is

^c^The TOP is the percentage of the top 2%/5%/10% candidates identified by the tools (except INVDOCK) to represent the prediction performance in the references cited, the higher the value of the TOP, the better the performance

^d^For HitPick, a sensitivity of 60.94%, a specificity of 99.99% and a precision of 92.11% is indicated in the references cited, normally, we can infer that the AUC of this tool is smaller than 0.61

^e^For INVDOCK, the TOP is the percentage of candidates identified by the tool, but the top percentage isn’t indicated in the references cited, the maximum value 50 is indicated

### Server usage and case study

**Fig. 4:**
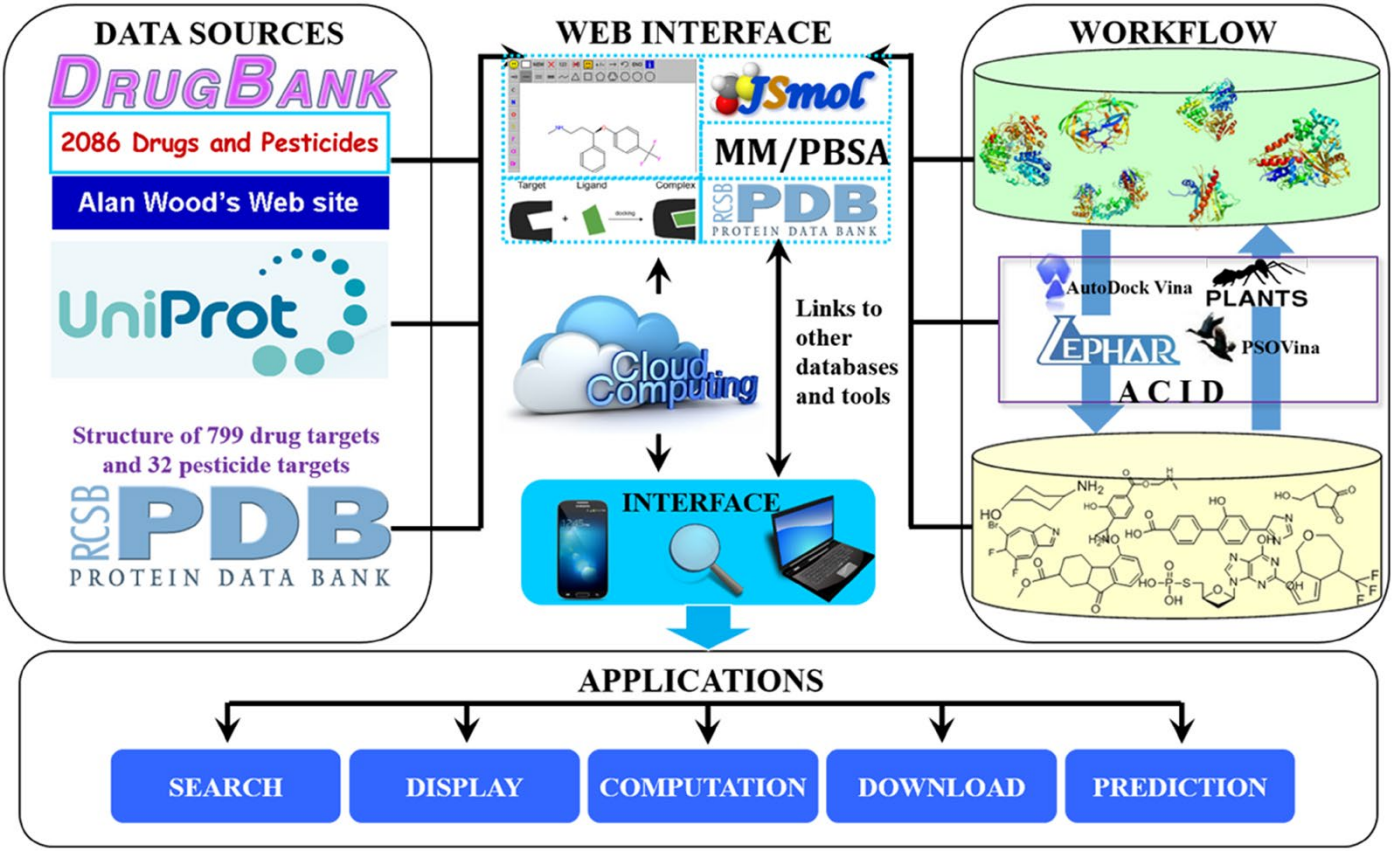
Schematic diagram describing data collection, integration, web interface, and applications of ACID web server

**Fig. 5:**
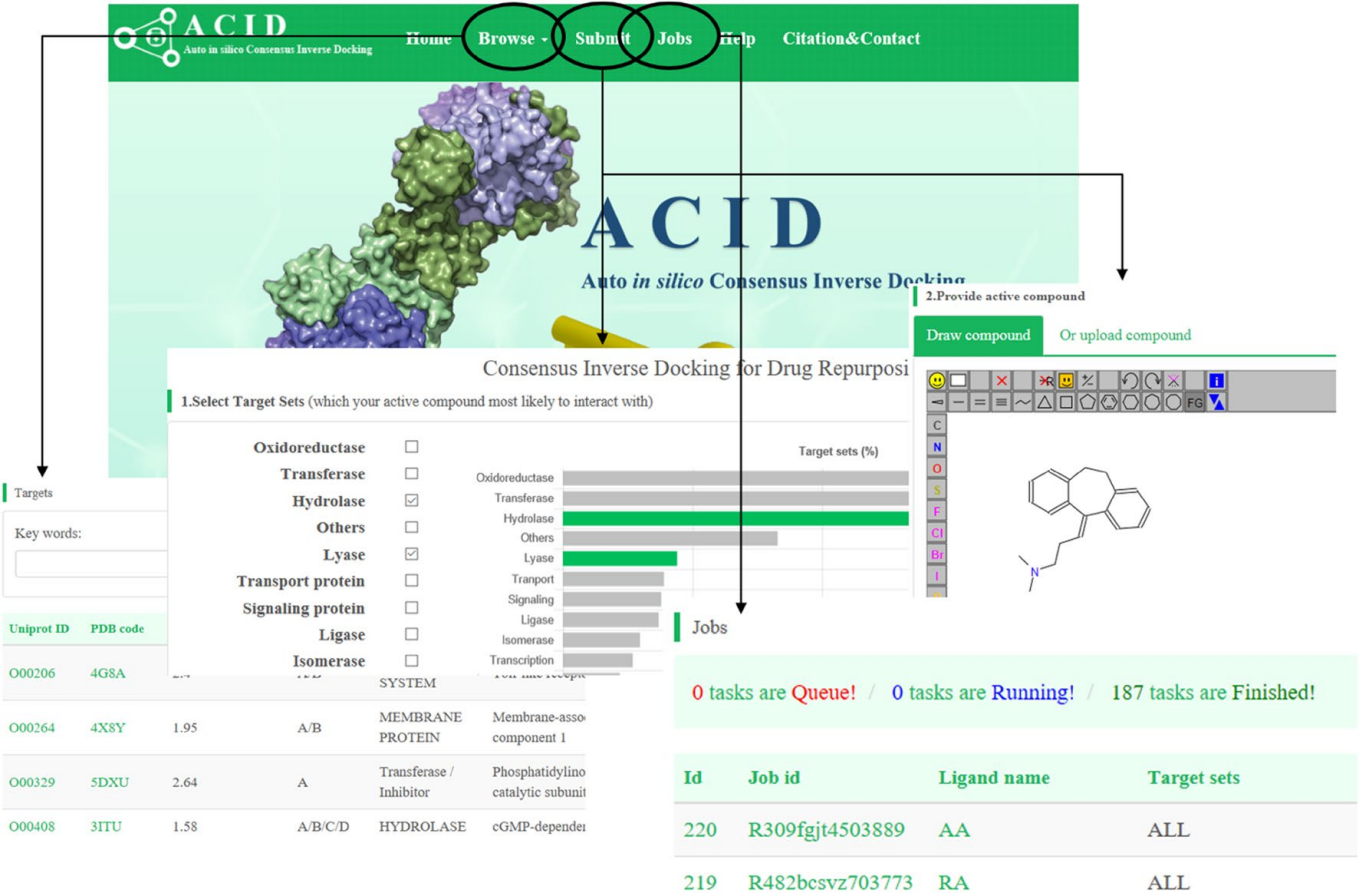
A screenshot montage of some usages of ACID. The screenshot of browse, submit, and jobs modules of ACID, including basic target-drug information, target classification, job submitting, and task management

In order to make online consensus inverse docking available, we build a public web server ACID. The bench-scientists can take advantage of the consensus inverse docking method. Data collection, integration, web interface, and applications of ACID were shown in Fig. 4. One can search a commercial drug automatically by entering keywords in the keyword search box, such as drug name, CAS no or InChI key. As shown in Fig. 5, the drug repurposing tasks can be submitted either by uploading or drawing molecule in the JSME plugin. Then, each ligand was converted into the Simplified Molecular Input Line Entry Specification (SMILES) representation by OpenBabel [45] tools (http://openbabel.sourceforge.net/). The 3D input of each ligand was produced from its SMILES using Corina (http://www.mol-net.de) [46]. In addition, the user need to customize target list from target database. An ID number is generated for the submitted job. The user can use the ID to check the status of the job on this web server. The binding models (in PDB format) of the molecule bound with the candidate targets can be downloaded through the “Download” hyperlink or browsed after clicking the “Show” hyperlink.

To evaluate the usage of the ACID web server, two examples are presented here. First, we show a test case using Citalopram as the query structure to find its potential target proteins via ACID server. Table S3 gives the results of predicted binding affinities. The protein target of Citalopram, namely sodium-dependent serotonin transporter (Rank 1, top 2%) as validated in the scientific report [47] are identified by ACID. Interestingly, the binding pose of Citalopram against sodium-dependent serotonin transporter with the rmsd of 0.98 Å compared to the crystal pose, indicating the reliability of this server. Another example shows that not only the intended targets could be identified by ACID, but also other proteins, leading to ‘off-target’ effects, which may have pharmacological consequences for drug repurposing. Amitriptyline is a classic medicine with multiple targets. The primary use is to treat a number of mental illnesses. It is particularly noteworthy that a very novel Amitriptyline repurposing is for treatment of triple-negative breast cancer by targeting on Poly (ADP-ribose) polymerase-1 (PARP1) [48], which is also predicted in the top 10% (rank 59) by ACID server. In addition, other uses include prevention of migraines, treatment of neuropathic pain such as fibromyalgia and postherpetic neuralgia.

## Conclusion

At the end of block-buster era for drug discovery, drug repurposing is a promising approach to address the ‘productivity gap’ that the global pharmaceutical giants are currently facing, which will improve the drug-discovery productivity. Inverse docking is proving to be a powerful tool for drug repurposing, which involves docking a drug in the potential binding cavities of a set of clinically relevant macromolecular targets. The critical issues related to inverse docking part are the prediction of correct binding pose and the estimation of some measure of the binding affinity. We have evaluated of several docking methods for inverse docking applications since the effectiveness of these methods in multiple target identification is unclear. A consensus inverse docking protocol was developed, which has a ~ 10% enhancement in success rate compared with the best single docking algorithm. Finally, an comprehensive web platform with a user-friendly interface was designed based on this protocol for drug repurposing to significantly reduce user time for data gathering and multi-step analysis without human intervention, which consists of the following three tools: (i) an automated consensus inverse docking workflow program, (ii) a compound database containing 2086 approved drugs with original therapeutic information, (iii) a known target database containing 831 protein structures from PDB covering 30 therapeutic areas. Differentiated with other tools, ACID outperforms other standalone algorithm in a better accuracy and more efficient way in summary.

## Supplementary information

**Additional file 1.** Additional tables and figures.

## Data Availability

All source code is available under open licenses on GitHub repository: https://github.com/fwangccnu/ACID. All datasets of this study can be downloaded under open licenses from the download page of the free web server http://chemyang.ccnu.edu.cn/ccb/server/ACID.

## Abbreviations

ADP-ribose: adenosine diphosphate ribose
AUC: area under the ROC curve
CAS: chemical abstracts service
CID: consensus inverse docking
JSME: JavaScript Molecule Editor
InChI: international chemical identifier
MM/PBSA: molecular mechanics/Poisson–Boltzmann surface are
NIH: The National Institutes of Health
PDB: protein data bank
NMR: nuclear magnetic resonance
PDTD: potential drug target database
RMSD: root-mean-square deviation
ROC: receiver operating characteristic curve
SMILES: simplified molecular input line entry specification
