# Supplementary material for: ACID: a free tool for drug repurposing using consensus inverse docking strategy
Source: J Cheminform. 2019 Nov 27;11:73. doi: 10.1186/s13321-019-0394-z (PMC6882193; doi:10.1186/s13321-019-0394-z)
Supplement: Supplementary file 1 — Additional file 1. Additional tables and figures. [file 13321_2019_394_MOESM1_ESM.doc]

**Additional Material**

**ACID: a free tool for drug repurposing using consensus inverse docking strategy**

Fan Wang^1,2†^, Feng-Xu Wu^1,2†^, Cheng-Zhang Li^1,2^, Chen-Yang Jia^1,2^, Sun-Wen Su^1,2^, Ge-Fei Hao^1,2,3,*^, and Guang-Fu Yang^1,2,4,*^

*^1^Key Laboratory of Pesticide & Chemical Biology, Ministry of Education, College of Chemistry, Central China Normal University, Wuhan 430079, P.R.China; ^2^International Joint Research Center for Intelligent Biosensor Technology and Health, Central China Normal University，Wuhan, 430079, China;* *^3^State Key Laboratory Breeding Base of Green Pesticide and Agricultural Bioengineering, Key Laboratory of Green Pesticide and Agricultural Bioengineering, Ministry of Education, Research and Development Center for Fine Chemicals, Guizhou University, Guiyang 550025, P. R. China; ^4^Collaborative Innovation Center of Chemical Science and Engineering, Tianjing 300072, P.R.China*

—————————

^†^Co-first authors.

*To whom correspondence should be addressed. *E-mail:* [*gfyang@mail.ccnu.edu.cn*](mailto:gfyang@mail.ccnu.edu.cn)*;* [*gfhao@mail.ccnu.edu.cn*](mailto:gfhao@mail.ccnu.edu.cn)

**Table S1** Conformation searching algorithms and scoring functions of docking softwares

| **Method** | **Conformation Sampling** | **Scoring Function** |
| --- | --- | --- |
| **AutoDock4.2** | Lamarckian genetic algorithm | Force field-based |
| **Vina1.1.2** | Iterated local search global optimizer | Physics-based |
| **DOCK6.6** | Geometric shape-matching algorithm | Force field-based |
| **PLANTS1.2** | Ant colony optimization | Empirical |
| **POSVina** | Particle swarm optimization | Physics-based |
| **LeDock** | Annealing evolution algorithm | Knowledge-based |
| **Gold3.2** | Genetic algorithms | goldscore: Force field-based  chemscore: Empirical  ASP: Knowledge-based |


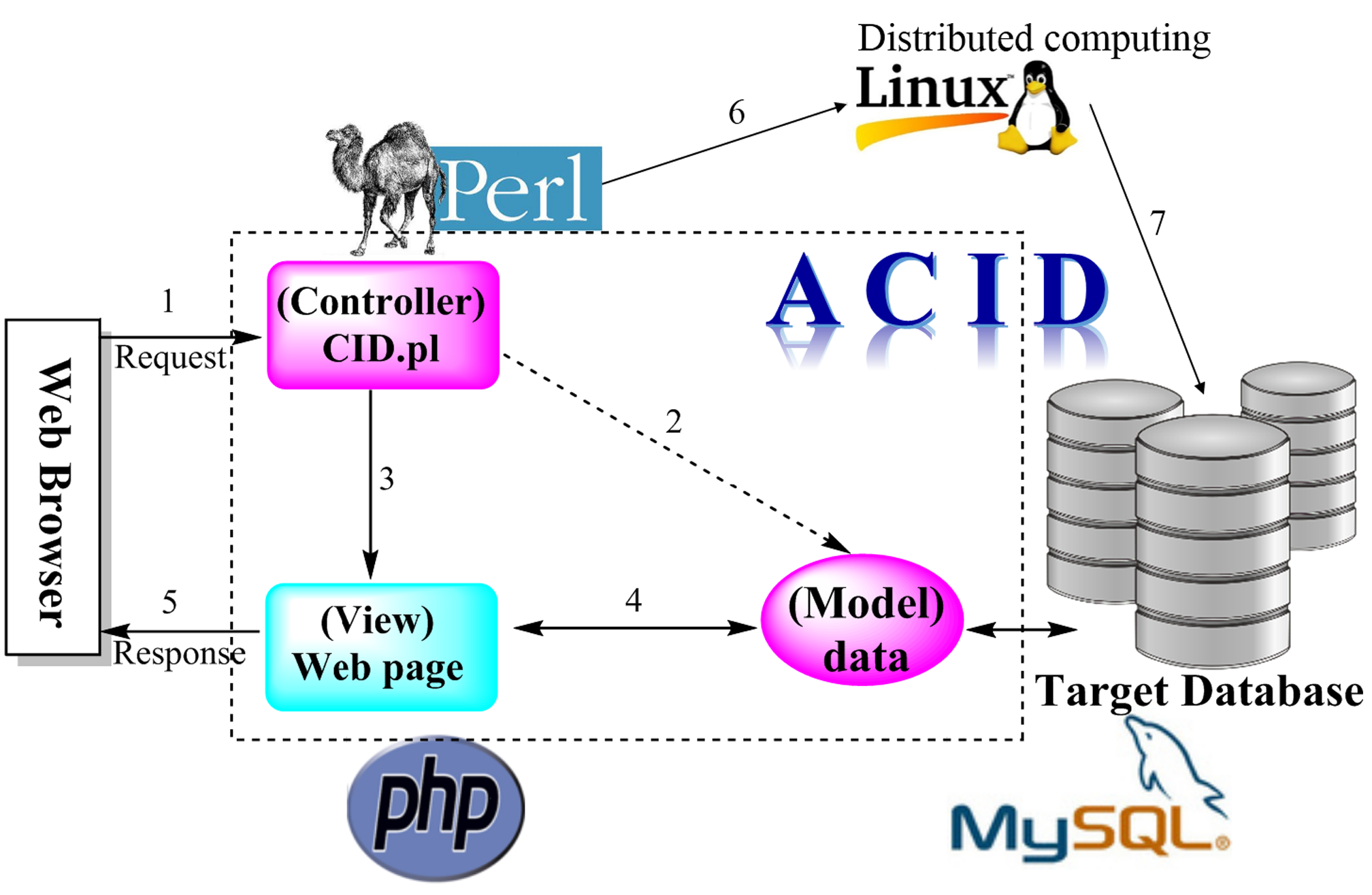
 **Figure S1** The Model-View-Controller design scheme of ACID web server


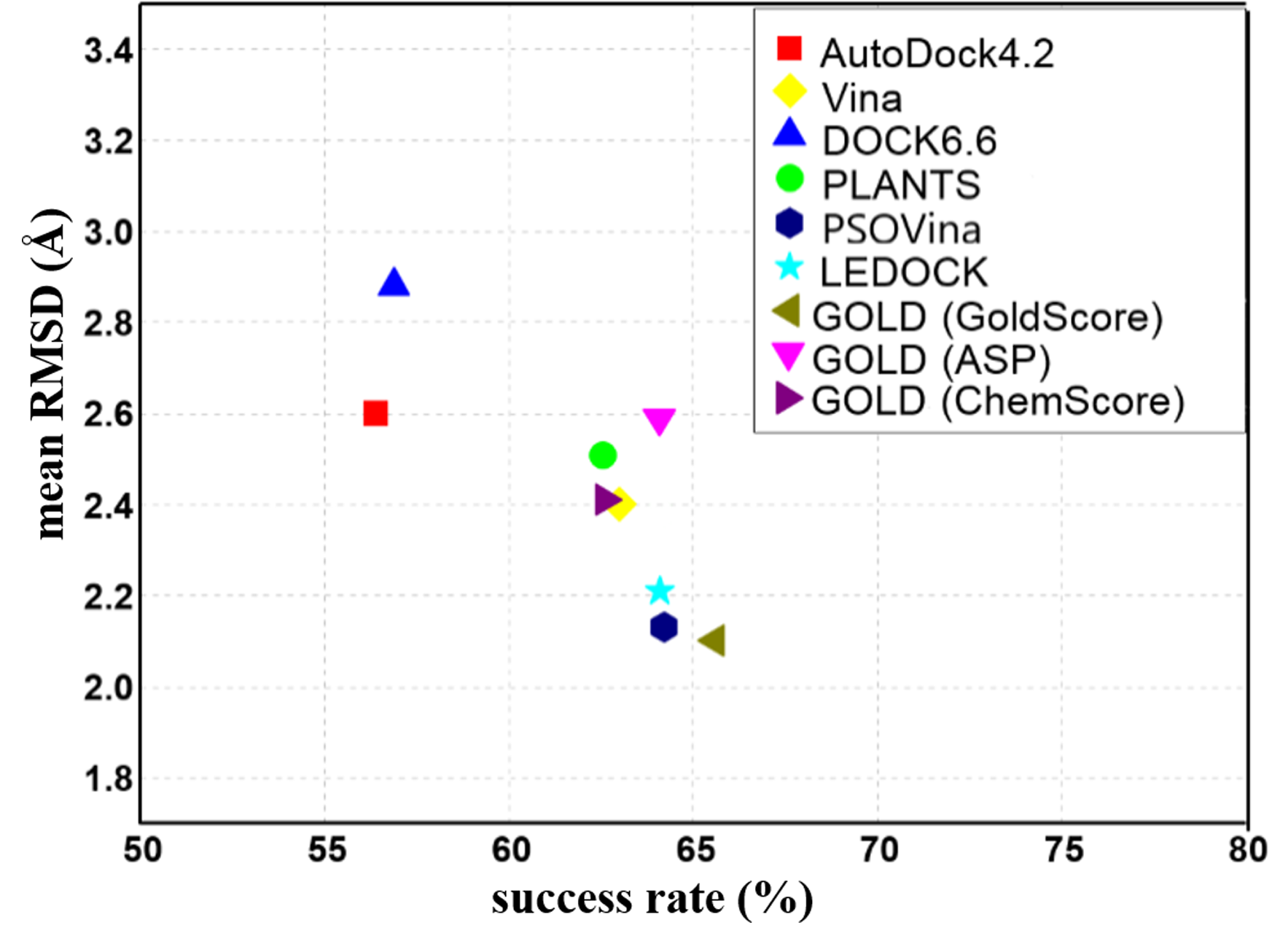


**Figure S2** Pose prediction performance of different docking method


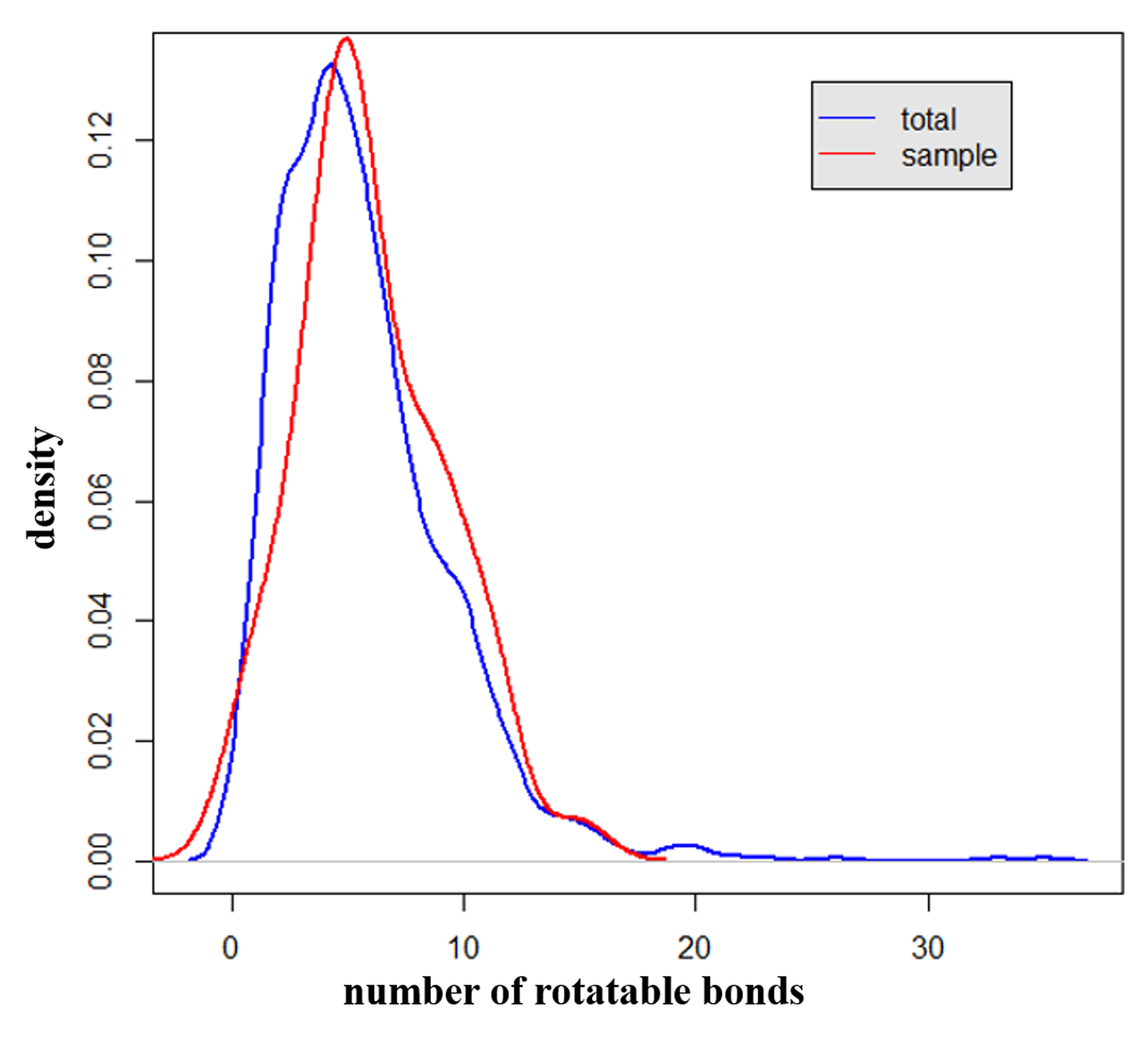


**Figure S3** The distribution of total and sampled drugs according to rotatable bonds

**Table S2** Predicting results for 51 randomly selected commercial drugs.

| Drug Name | rotate | Mol. Weigh. | 2% | 5% | 10% |
| --- | --- | --- | --- | --- | --- |
| Tetrahydrofolic acid | 9 | 445.429 | TRUE |  |  |
| L-Histidine | 3 | 155.155 |  | TRUE |  |
| Riboflavin | 5 | 376.364 |  |  | TRUE |
| Folic Acid | 9 | 441.397 |  | TRUE |  |
| Vitamin A | 5 | 286.452 | TRUE |  |  |
| Vitamin E | 12 | 430.706 | TRUE |  |  |
| Ramipril | 11 | 416.511 |  |  | TRUE |
| Nicotine | 1 | 162.232 | TRUE |  |  |
| Cevimeline | 0 | 199.313 |  | TRUE |  |
| Pyrimethamine | 2 | 248.711 | TRUE |  |  |
| Adapalene | 4 | 412.52 | TRUE |  |  |
| Citalopram | 6 | 324.392 | TRUE |  |  |
| Eletriptan | 6 | 382.519 |  |  | TRUE |
| Moxifloxacin | 4 | 401.431 |  | TRUE |  |
| Oxyphenonium | 9 | 348.5 | TRUE |  |  |
| Isoetarine | 5 | 239.311 | TRUE |  |  |
| Temazepam | 1 | 300.74 | TRUE |  |  |
| Benzatropine | 4 | 307.429 | TRUE |  |  |
| Ziprasidone | 4 | 412.936 | TRUE |  |  |
| Metoprolol | 9 | 267.364 |  |  | TRUE |
| Ropinirole | 7 | 260.375 |  |  | TRUE |
| Disopyramide | 8 | 339.474 | TRUE |  |  |
| Atomoxetine | 6 | 255.355 |  | TRUE |  |
| Zolmitriptan | 5 | 287.357 | TRUE |  |  |
| Amitriptyline | 3 | 277.403 | TRUE |  |  |
| Indomethacin | 5 | 357.788 |  |  | TRUE |
| Buclizine | 6 | 433.028 |  | TRUE |  |
| Doxylamine | 6 | 270.369 |  | TRUE |  |
| Aripiprazole | 7 | 448.385 |  | TRUE |  |
| Ethopropazine | 5 | 312.472 | TRUE |  |  |
| Nimodipine | 10 | 418.44 |  | TRUE |  |
| Promazine | 4 | 284.419 | TRUE |  |  |
| Hyoscyamine | 5 | 289.369 |  | TRUE |  |
| Dipivefrin | 9 | 351.437 |  |  | TRUE |
| Tamoxifen | 8 | 371.515 | TRUE |  |  |
| Mefloquine | 4 | 378.312 |  |  | FALSE |
| Biotin | 5 | 244.311 |  |  | FALSE |
| Pyridoxal | 2 | 167.162 |  |  | FALSE |
| Dihomo-γ-linolenic acid | 15 | 306.483 |  |  | FALSE |
| Pravastatin | 11 | 424.528 |  |  | FALSE |
| Valsartan | 11 | 435.519 |  |  | FALSE |
| Carbidopa | 4 | 226.229 |  |  | FALSE |
| Tramadol | 4 | 263.375 |  |  | FALSE |
| Betaxolol | 11 | 307.428 |  |  | FALSE |
| Oseltamivir | 8 | 312.405 |  |  | FALSE |
| Lovastatin | 7 | 404.54 |  |  | FALSE |
| Nevirapine | 1 | 266.298 |  |  | FALSE |
| Sulfisoxazole | 3 | 267.304 |  |  | FALSE |
| Isradipine | 6 | 371.387 |  |  | FALSE |
| Olmesartan | 8 | 446.502 |  |  | FALSE |
| Amsacrine | 5 | 393.459 |  |  | FALSE |

**Table S3** Detailed predicting results for 35 randomly selected commercial drugs.

| drugs | pdb | target | MM/PBSA | | Xscore | |
| --- | --- | --- | --- | --- | --- | --- |
|  |  |  | ΔG_bind_  kcal/mol | rank | ΔG_bind_  kcal/mol | rank |
| Tetrahydrofolic acid | 1WSV | Aminomethyltransferase, mitochondrial | -2.78 | 225 | -8.49 | 207 |
|  | 2CFI | 10-FTHFDH | -12.93 | 89 | -9.45 | 29 |
|  | 3OU5 | Serine methylase | -7.49 | 169 | -7.64 | 342 |
|  | 4CCZ | Methionine synthase | -24.77 | 8 | -8.21 | 248 |
|  | 5TC4 | Methenyltetrahydrofolate cyclohydrolase | -4.98 | 204 | -8.66 | 163 |
| L-Histidine | 4E1O | Histidine decarboxylase | -13.73 | 13 | -6.21 | 38 |
|  | 4G84 | Histidine--tRNA ligase, cytoplasmic | -6.94 | 37 | -5.84 | 79 |
| Riboflavin | 1NB0 | Riboflavin kinase | -40.64 | 15 | -8.29 | 235 |
|  | 1PKV | Riboflavin synthase | -17.82 | 776 | -6.98 | 869 |
| Folic Acid | 4LRH | Folate receptor alpha | -50.33 | 1 | -9.89 | 18 |
|  |  |  |  |  |  |  |
| Vitamin A | 3HX3 | Retinaldehyde-binding protein 1 | -34.11 | 13 | -9.91 | 14 |
|  | 3O4R | NRDR | -15.81 | 119 | -9.36 | 66 |
|  | 4WP7 | Retinal dehydrogenase 1 | -30.84 | 26 | -9.35 | 69 |
|  | 4X2Q | Retinal dehydrogenase 2 | -15.39 | 231 | -8.75 | 170 |
|  | 5FHZ | Retinaldehyde dehydrogenase 3 | -29.29 | 32 | -9.24 | 86 |
|  | 5HBS | Retinol-binding protein 1 | -37.61 | 3 | -9.9 | 15 |
| Vitamin E | 1NRL | Orphan nuclear receptor PAR1 | -34.17 | 115 | -10.9 | 53 |
|  | 4I5L | PP2A-alpha | -29.82 | 200 | -7.89 | 568 |
|  | 4OMJ | SEC14-like protein 2 | -54.88 | 2 | -11.8 | 8 |
|  | 4RA4 | Protein kinase C alpha type | -24.91 | 327 | -9.71 | 252 |
|  | 4TLG | SEC14-like protein 4 | -47.43 | 14 | -11.84 | 7 |
|  | 4UYB | SEC14-like protein 3 | -42.2 | 35 | -11.26 | 28 |
| Ramipril | 5AMC | Angiotensin-converting enzyme | -13.16 | 78 | -8.7 | 385 |
|  |  |  |  |  |  |  |
| Nicotine | 2FY3 | Choline O-acetyltransferase | -10.58 | 95 | -7.1 | 100 |
|  | 4UXU | NACHR alpha-9 | -12.21 | 58 | -6.48 | 443 |
|  | 4ZK4 | Neuronal acetylcholine receptor subunit alpha-3 | -26.73 | 1 | -7.37 | 13 |
|  | 5AFN | Neuronal acetylcholine receptor subunit alpha-7 | -10.35 | 98 | -6.77 | 289 |
|  | 2YK1 | anti-nicotine Fab fragment | -21.56 | 3 | -9.31 | 1 |
| Cevimeline | 5CXV | Muscarinic acetylcholine receptor M1 | -6.98 | 67 | -7.45 | 8 |
|  |  |  |  |  |  |  |
| Pyrimethamine | 1J3K | Bifunctional dihydrofolate reductase-thymidylate synthase | -17.43 | 88 | 8.26 | 38 |
|  | 1KMV | Dihydrofolate reductase | -24.56 | 11 | -8.09 | 74 |
| Adapalene | 1FCY | Retinoic acid receptor gamma | -44.92 | 3 | -12.58 | 1 |
|  | 2GL8 | Retinoid X receptor gamma | -12.53 | 75 | -8.23 | 46 |
|  | 2P1T | Retinoic acid receptor RXR-alpha | -34.79 | 4 | -12.07 | 4 |
|  | 3KMR | Retinoic acid receptor alpha | -55.3 | 1 | -12.2 | 3 |
|  | 4DM6 | Retinoic acid receptor beta | -54.36 | 2 | -12.44 | 2 |
|  | 5HJP | Retinoic acid receptor RXR-beta | -31.86 | 5 | -11.77 | 6 |
| Citalopram | 5I71 | Sodium-dependent serotonin transporter | -38.28 | 1 | -9.78 | 1 |
|  |  |  |  |  |  |  |
| Eletriptan | 4IAR | 5-hydroxytryptamine receptor 1B | -8.75 | 297 | -8.75 | 314 |
|  | 4IB4 | 5-hydroxytryptamine receptor 2B | -16.89 | 80 | -9.16 | 165 |
| Moxifloxacin | 1ZXM | DNA topoisomerase 2-alpha | 52.83 | 36 | -9.05 | 2 |
|  |  |  |  |  |  |  |
| Oxyphenonium | 5CXV | Muscarinic acetylcholine receptor M1 | -43.99 | 1 | -9.96 | 1 |
|  |  |  |  |  |  |  |
| Isoetarine | 2RH1 | Beta-2 adrenergic receptor | -16.61 | 4 | -7.35 | 19 |
|  |  |  |  |  |  |  |
| Temazepam | 4COF | Gamma-aminobutyric acid receptor subunit beta-3 | -19.98 | 20 | -9.29 | 3 |
|  |  |  |  |  |  |  |
| Benzatropine | 5CXV | Muscarinic acetylcholine receptor M1 | -28.38 | 2 | -10.07 | 2 |
|  |  |  |  |  |  |  |
| Ziprasidone | 4IAR | 5-hydroxytryptamine receptor 1B | -26.07 | 18 | -9.11 | 31 |
|  | 5AER | D(2) dopamine receptor | -20.5 | 46 | -7.82 | 160 |
|  | 5CXV | Muscarinic acetylcholine receptor M1 | -27.5 | 13 | -9.65 | 12 |
|  | 5DSG | Muscarinic acetylcholine receptor M4 | -13.16 | 124 | -9.73 | 10 |
| Metoprolol | 2RH1 | Beta-2 adrenergic receptor | -23.58 | 77 | -7.83 | 116 |
|  |  |  |  |  |  |  |
| Ropinirole | 3PBL | D(3) dopamine receptor | -23.54 | 49 | -8.39 | 105 |
|  | 4IAR | 5-hydroxytryptamine receptor 1B | -23.59 | 47 | -8.09 | 240 |
|  | 4IB4 | 5-hydroxytryptamine receptor 2B | -27.74 | 21 | -8.35 | 118 |
| Disopyramide | 1S1G | Potassium voltage-gated channel subfamily D member 3 | -27.33 | 39 | -9.28 | 151 |
|  | 5CXV | Muscarinic acetylcholine receptor M1 | -38.58 | 6 | -10.42 | 6 |
| Atomoxetine | 5H8Q | Glutamate receptor ionotropic, NMDA 1 | -24.59 | 29 | -8.95 | 30 |
|  |  |  |  |  |  |  |
| Zolmitriptan | 4IAR | 5-hydroxytryptamine receptor 1B | -26.26 | 3 | -7.96 | 7 |
|  |  |  |  |  |  |  |
| Amitriptyline | 1S1G | Potassium voltage-gated channel subfamily D member 3 | -18.8 | 58 | -8.3 | 165 |
|  | 3APV | AGP 2 | -21.78 | 31 | -9.53 | 8 |
|  | 4DJH | Kappa-type opioid receptor | -26.55 | 9 | -8.94 | 48 |
|  | 4IAR | 5-hydroxytryptamine receptor 1B | -21.83 | 30 | -8.81 | 66 |
|  | 4N6H | Delta-type opioid receptor | -20.74 | 41 | -8.31 | 162 |
|  | 4PMP | Tyrosine kinase receptor A | 2.74 | 420 | -8.43 | 137 |
|  | 5CXV | Muscarinic acetylcholine receptor M1 | -31.65 | 4 | -9.56 | 7 |
|  | 5DSG | Muscarinic acetylcholine receptor M4 | -19.65 | 80 | -9.62 | 5 |
|  | 5HK1 | Sigma non-opioid intracellular receptor 1 | -40.24 | 1 | -9.65 | 3 |
|  | 5HA9 | Poly [ADP-ribose] polymerase 1 | -18.98 | 59 | -8.80 | 67 |
|  | 5J03 | Potassium voltage-gated channel subfamily KQT member 3 | -16.61 | 92 | -7.48 | 169 |
| Indomethacin | 2ZB4 | Prostaglandin reductase 2 | -25.71 | 64 | -9.19 | 98 |
|  | 3B1M | Peroxisome proliferator-activated receptor gamma | -24.82 | 83 | 8.96 | 151 |
|  | 3VI8 | Peroxisome proliferator-activated receptor alpha | -12.99 | 402 | -8.79 | 203 |
| Buclizine | 5CXV | Muscarinic acetylcholine receptor M1 | -24.04 | 15 | -9.43 | 31 |
|  |  |  |  |  |  |  |
| Doxylamine | 5CXV | Muscarinic acetylcholine receptor M1 | -27.34 | 11 | -8.66 | 20 |
|  |  |  |  |  |  |  |
| Ethopropazine | 5CXV | Muscarinic acetylcholine receptor M1 | -29.87 | 9 | -9.45 | 33 |
|  |  |  |  |  |  |  |
| Nimodipine | 2F3Y | Voltage-dependent L-type calcium channel subunit alpha-1C | -35.44 | 65 | -7.81 | 569 |
|  | 2VAY | Voltage-dependent L-type calcium channel subunit alpha-1S | -25.24 | 365 | -7.29 | 823 |
| Promazine | 4PF3 | Mineralocorticoid receptor | -44.48 | 10 | -9.64 | 33 |
|  | 5AER | D(2) dopamine receptor | -15.13 | 289 | -6.78 | 916 |
|  | 5CXV | Muscarinic acetylcholine receptor M1 | -24.3 | 44 | -8.78 | 19 |
| Hyoscyamine | 5CXV | Muscarinic acetylcholine receptor M1 | -31.43 | 3 | -8.95 | 20 |
|  | 5DSG | Muscarinic acetylcholine receptor M4 | -25.33 | 28 | -8.96 | 18 |
| Dipivefrin | 1P0I | Cholinesterase | -8.02 | 567 | -7.47 | 855 |
|  | 2RH1 | Beta-2 adrenergic receptor | -29.97 | 17 | -9.51 | 44 |
| Tamoxifen | 3CBP | Estrogen receptor | -6.96 | 104 | -9.01 | 123 |
|  | 3OLL | Estrogen receptor beta | -30.58 | 10 | -10.7 | 5 |
|  | 4RA4 | Protein kinase C alpha type | -21.8 | 40 | -9.21 | 68 |
| Aripiprazole | 3PBL | D(3) dopamine receptor | -22.74 | 51 | -9.68 | 20 |
|  | 4IAR | 5-HT1B | -16.41 | 123 | -9.6 | 26 |
|  | 5AER | D(2) dopamine receptor | -23.08 | 49 | -8.44 | 98 |
|  | 5CXV | Muscarinic acetylcholine receptor M1 | -16.8 | 119 | -9.87 | 17 |
|  | 5DSG | Muscarinic acetylcholine receptor M4 | -0.91 | 208 | -10.01 | 13 |
